# Organoid technology for personalized pancreatic cancer therapy

**DOI:** 10.1007/s13402-021-00585-1

**Published:** 2021-01-25

**Authors:** Axel Bengtsson, Roland Andersson, Jonas Rahm, Karthik Ganganna, Bodil Andersson, Daniel Ansari

**Affiliations:** Department of Surgery, Clinical Sciences Lund, Skåne University Hospital, Lund University, Skåne University Hospital, Lund, SE-221 85 Lund, Sweden

**Keywords:** Pancreatic cancer, Organoids, Personalized medicine, Drug screening

## Abstract

**Background:**

Pancreatic ductal adenocarcinoma has the lowest survival rate among all major cancers and is the third leading cause of cancer-related mortality. The stagnant survival statistics and dismal response rates to current therapeutics highlight the need for more efficient preclinical models. Patient-derived organoids (PDOs) offer new possibilities as powerful preclinical models able to account for interpatient variability. Organoid development can be divided into four different key phases: establishment, propagation, drug screening and response prediction. Establishment entails tailored tissue extraction and growth protocols, propagation requires consistent multiplication and passaging, while drug screening and response prediction will benefit from shorter and more precise assays, and clear decision-making tools.

**Conclusions:**

This review attempts to outline the most important challenges that remain in exploiting organoid platforms for drug discovery and clinical applications. Some of these challenges may be overcome by novel methods that are under investigation, such as 3D bioprinting systems, microfluidic systems, optical metabolic imaging and liquid handling robotics. We also propose an optimized organoid workflow inspired by all technical solutions we have presented.

## Introduction

Pancreatic ductal adenocarcinoma is a highly aggressive tumor type with a 5-year survival rate below 5% [1]. It currently ranks as the third leading cause of cancer-mortality, but is expected to become the second leading cause in the U.S.A. by 2030 [2, 3]. The low and non-durable response rate to current treatments is attributed to the inherent aggressive nature of pancreatic cancer cells, the dense desmoplastic reaction and the molecular heterogeneity observed in this tumor type. In addition, most pancreatic cancers appear to be non-immunogenic, explaining the refractoriness to immunotherapeutic compounds [4, 5]. Phase III drug trials are plagued by high dropout rates and low initial clinical correlations, explaining the limited success rate [6]. If interpatient biological variability, as evident by the characterization of the molecular subtypes [7] and long-term survivors of the disease [8], is to be addressed as well, development of viable models for personalized medicine is next in line. Personalized medicine, or precision medicine, is an approach for improving the outcome of cancer therapies by grouping patients based on their predicted individual responses to therapy. Limitations of classical preclinical models, such as 2D monolayer cell cultures, genetically engineered mouse models, and patient-derived tumor xenografts, include a lack of comparability with normal tissue [9, 10], culturing times exceeding months [11], foreign stromal components [12, 13] and selection of more aggressive phenotypes [14, 15].

**Fig. 1:**
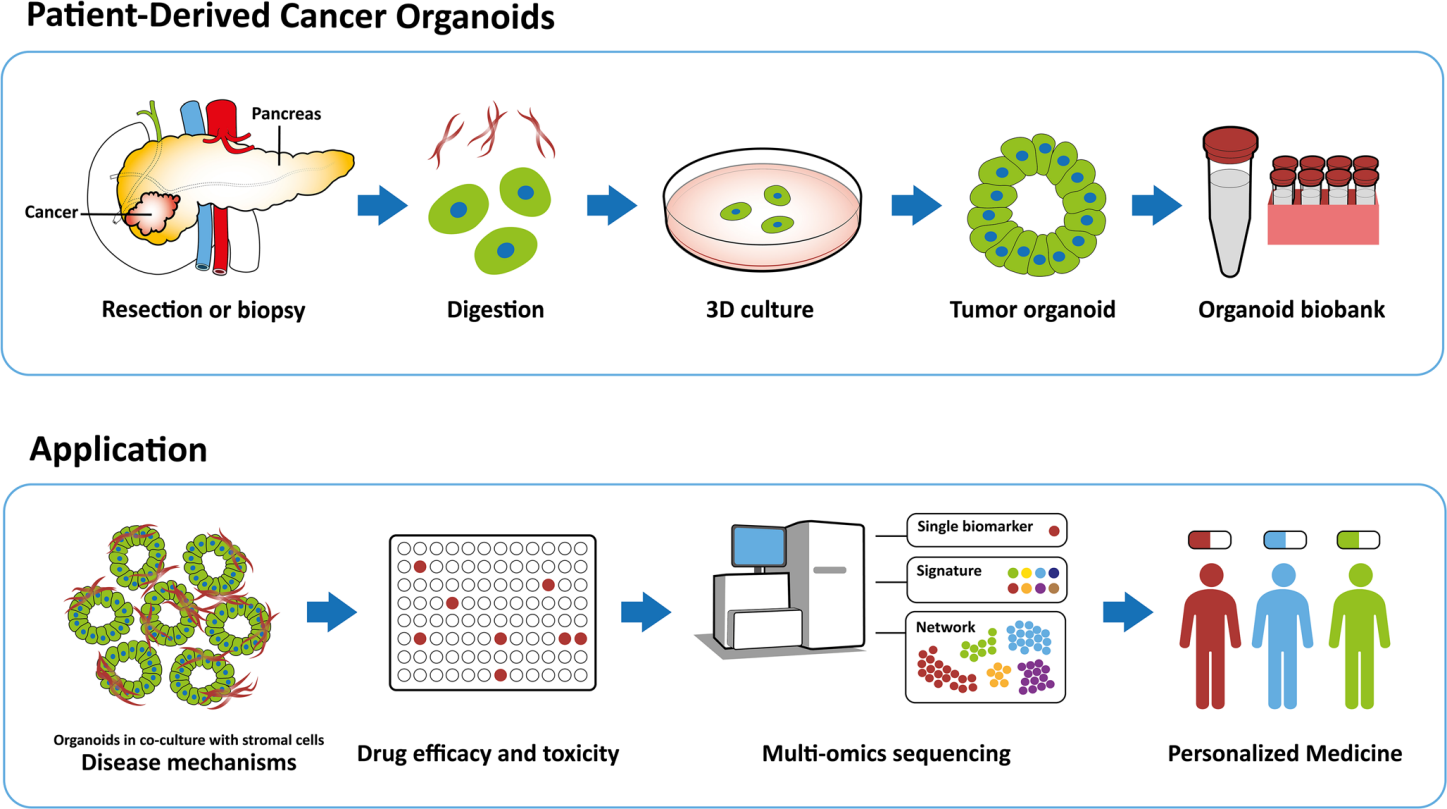
Generation and application of patient-derived pancreatic cancer organoids

**Fig. 2:**
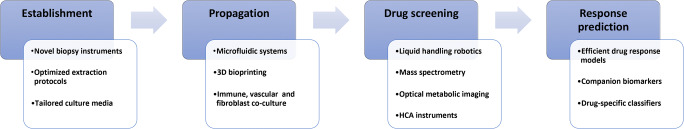
Combining organoid methodology and recent developments in biotechnology as part of a personalized medicine workflow for pancreatic cancer. HCA, high-content analysis

Conversely, patient-derived organoids (PDOs) can be generated from extracted patient tumor tissue (primary tumor and metastases) within two to four weeks following surgery or biopsy via endoscopic ultrasound [16, 17] (Fig. 1). PDOs recapitulate histopathologic and genomic profiles of the tissue of origin while maintaining genomic stability throughout passaging [18–20]. Organoids represent cell cultures kept in an organ-type specific matrix for 3-dimensional growth by avoiding cell attachment to the surrounding plate. 3D cultures derived from established monolayer cell lines are called spheroids, whereas patient-derived organoids are defined as multicellular extracellular matrix (ECM)-dependent units derived directly from patient tissue. The extracted tissue is first minced, then enzymatically digested and placed in a matrix (collagen or Matrigel) together with a culture medium containing growth factors and modulators enriching epithelial cells. The cells form microscopically visible organ-like structures within two days, with passaging and propagation ready once 80% confluency is reached [13, 17]. Organoids have been used to simulate a variety of diseases. Intestinal organoids, for instance, have been utilized for the study of cystic fibrosis, inflammatory bowel disease and *Clostridium difficile* infection. Cancer has been studied utilizing organoids in the context of neoplastic transformation and therapeutic screening, and pancreatic cancer is at the forefront of organoid technology applications. However, there are major challenges pertaining to the implementation of organoid technology in personalized pancreatic cancer care. These challenges can be divided into different phases of the organoid workflow process, as depicted in Fig. 2. The establishment phase entails extraction of tumor tissue and initial culturing. Here, ensuring a good neoplastic cellularity is key for a successful initial outgrowth, a challenge highlighted by Tiriac and colleagues in a recent multicenter study [17]. While resection has remained the main extraction method, introducing novel biopsy techniques may broaden potential therapeutic applications. The next phase, termed ‘propagation’, describes the conditions in which the neoplastic cell clusters can multiply for further passaging. There are various culture methods currently in use to minimize deviation from the in vivo material. The third and fourth phases encompass different approaches to therapeutic assays of promising novel compounds. Drug screening in the in vitro setting has traditionally consisted of a short phase of cell line screening and the effect measures of half maximal inhibitory concentration (IC_50_). With the advent of experimental therapeutic organoid assays, more complex yet faster assays are under development. These powerful viability assays, together with drug response models and drug-specific classifiers could provide the basis for rapid therapeutic decision-making.

This review will focus on the applications of PDOs for high-throughput drug screening and personalized therapy in pancreatic cancer, utilizing the previously outlined development steps of establishment, propagation, drug screening and response prediction. The review will conclude with a proposed organoid workflow and a remark on the potential for organoids to be introduced in the pipeline of promising chemotherapeutics.

## Current organoid models

3D cell culture started with the hanging drop tissue method developed by Ross Harrison in 1906 [21]. In 1977, the study of the extracellular matrix of chondrosarcoma led to the development of an ECM substrate with basal membrane characteristics, today known as Matrigel [22]. This enabled the characterization of ECM proteins and their role in tissue morphogenesis. The parallel progress of stem cell biology led in 2003 to the discovery that progenitor cells generated complex structures resembling the tissue of origin when embedded in Matrigel [23]. The Clevers laboratory pioneered culturing methods for epithelial organoids with cells from a variety of organs, even without stem cell markers, starting in the early 2000s. In 2013, the laboratory grew budding cyst-like structures from pancreatic duct cells using the Wnt-Lgr5-Rspo signaling axis [24]. In 2015 the first patient-derived pancreatic tumor organoid model was published as a result of a collaboration between the Clevers and Tuveson laboratories. This protocol includes Wnt-activating serum-free media over a dome of Matrigel [13].

Other methods for establishing pancreatic cancer organoids have followed. The Muthuswamy lab cultured cells in an overlay over a Matrigel bed without Wnt-signaling medium [25], while the Skala group developed a PDO culture model with visible innate fibroblasts by initially growing them in mice [26]. In 2019, the first comprehensive protocol for establishing pancreatic cancer organoids from resected tissue was published by Baker et al. [16] (Clevers and Tuveson groups). This protocol enables long-term propagation of PDOs and has, in a slightly modified form, been employed for high-throughput screening (HTS) of drugs [20, 27]. A limitation of this model is the absence of cancer-associated fibroblasts (CAFs) after a few days in culture media. Another research group maintained normal pancreatic tissue organoids in collagen matrices and Transwell cell culture plates with an air-liquid interface (ALI) [28]. At this stage, the ALI method is mainly used to produce kidney and intestinal organoids [29], whereas the use of collagen-based hydrogel may provide a solution for automated processes like bioprinting [30].

## Organoid establishment

To construct a robust personalized therapy workflow for use in the clinic, novel biopsy technologies have to be combined with optimized extraction and establishment protocols. This will increase tumor cell viability, minimize normal cell contamination and increase establishment rates. The first patient-derived tumor pancreatic model by the Clevers and Tuveson laboratories showed establishment rates of 75% and 83%, respectively. With the use of fine-needle aspiration, Tuveson and colleagues achieved an 87% success rate, however only 66% of organoids survived the fifth passage [17]. According to the authors, a success rate goal of at least 90% should be set for clinical implementation [17]. While the lack of a tailored medium composition is thought to contribute to the failure of growth in some organoids, the enrichment of distinctive subclones is also a cause for concern. The selection of specific protein-dependent pancreatic cancer subclones without identifiable genetic mutations has been described [27, 31], showing an unmet need to define ideal media compositions for specific tumor types.

Obviously, success rates vary between extraction methods. In addition to novel biopsy techniques, other forms of tumor cell extraction have gained interest in recent years. Circulating tumor cells (CTCs) are primary tumor and/or metastatic cells shed into the blood stream to seed metastatic lesions. Among many differing technical solutions for the separation of seeding-capable tumor cells from peripheral blood, Zhang et al. demonstrated that, with a microfluidic CTC-chip, brain-metastatic breast cancer cells were able to undergo 3D-coculturing with fibroblasts and extracellular matrix components on the same chip and to maintain strong mutational concordance [32].

## Organoid propagation

### 3D bioprinting systems

While ensuring a medium favorable to a specific tumor type is important for organoid establishment, ensuring homogeneity or a “level playing field” for seeded organoid cells may be equally important when opting for consistent drug screening results further down the organoid pipeline. Bioprinting entails controlled propulsion of cell-laden bio-ink onto culture plates to achieve a higher degree of consistency in the cell seeding and placement process [33]. At the current stage, only spheroids of pancreatic cancer have been printed. Hou and colleagues used the NanoShuttle® reagent containing nanoparticles of gold, iron oxide and poly-L-lysine to form pancreatic cancer spheroids over a magnetic drive without ECM compound [34]. The combination of established cell lines, with or without CAFs, garnered homogeneous spheres when plates were assessed using their high-resolution image processing instrument [34].

Bioprinting of actual patient-derived organoid models has been reported [30]. Glioblastoma and sarcoma PDOs have been used in conjunction with two commercially available bioprinters to develop a new technique called “immersion bioprinting”, a type of extrusion-based printing that preserves ECM compound and avoids adherence of the bio-ink to the well wall. Extrusion-based printing involves pneumatic or mechanical pressure to propel cell-laden bio-ink through a nozzle. A gelatin-bath and a special collagen-hyaluronic acid hydrogel formulation is used as the initial receiving substrate, while UV-light generates cross-linking reactions between thiol groups in the hydrogel. Afterwards, the gelatin-bath is aspirated, and proper cell culture medium is added, which allows the bio-ink to remain spherical in smaller HTS vessels. As a proof-of-principle, the inventors of immersion bioprinting tested three chemotherapeutic agents against available cultures in the study and were able demonstrate varying but positive dose-response trends in all PDOs [30].

Laser-assisted bioprinting (LAB) is a technology that brings higher resolution and speed than extrusion-based methods, making it suitable for automated high-throughput assays [33]. Utilizing LAB, Hakobyan et al. printed pancreatic exocrine spheroids from rat acinar cells for the study of early pancreatic tumorigenesis [35]. To this end, a pulsed infrared laser beam is focused onto a glass slide containing a thin slice of bio-ink. The slide is coated with a gold layer absorbing the beam energy to create cavitation-like bubbles that propel bio-ink droplets onto the hydrogel. Another gel layer is added on top, creating a matrix-sandwich. The authors embedded the spheroids in gelatin methacrylate-based collagen hydrogels for irradiation-induced cross-linking in order to adjust the mechanical properties of the matrix-sandwich. This optimized their cell encapsulation procedure, allowing for better control over cell density and positioning [35]. Bioprinting technology is still in its initial stages, but if the propagation process can be expanded to include both stromal and neoplastic cells, technologies such as LAB could offer a possibility for spatial arrangements of neoplastic cells and its CAFs at the single-cell level.

### Microfluidic systems and pancreatic stellate cells

There have been efforts at incorporating more factors that may infer drug resistive properties. Microfluidic systems control fluid perfusion, nutrient flow and tissue architecture to adjust for specific properties in pancreatic cancer (e.g. high intra-tumoral pressure and aberrant interstitial perfusion). Microfluidics with 3D model cultures are called “organ-on-a-chip” platforms. OrganoPlate is a high-throughput organ-on-a-chip platform developed by biotech company MIMETAS. The chips inside the platform contain a gel channel with ECM-embedded cells and two adjacent perfusion channels with medium. The interface can be placed on a rocking platform that generates different types of flow of media in relation to the gel channel. Kramer et al. investigated the effect of flow orientation on viability and compared the drug inhibitory concentrations between 2D cultures and 3D OrganoPlate cultures of a non-metastatic pancreatic cancer cell line [36]. Their platform consisted of a 384-well interface with a total of 40 chips. Different flow types generated different permeation patterns. Under interstitial flow, the spheroids showed a 3-fold lower sensitivity to gemcitabine than under the perfusion flow pattern [36]. In another study, patient-derived head-and-neck cancer organoid lines were tested on a bigger 96-chip OrganoPlate array, showing differing interpatient sensitivities to concentration series of cisplatin and niraparib [37]. Currently, a new platform named “organoid-on-a-chip” is being developed by Hubrecht Organoid Technology in collaboration with MIMETAS [38].

Pancreatic stellate cells (PSCs) are major contributors to the resilience of pancreatic cancer, as they make up more than half of pancreatic CAFs. To increase biomimicry, microfluidic systems can be combined with stromal key components. Lee et al. cocultured pancreatic tumor spheroids with PSCs in microchannels consisting of a collagen gel-solution [39]. F-actin and α-SMA staining showed spindle-shaped PSCs and elevated F-actin and α-SMA levels, demonstrating fibroblast activation. Proteome analysis revealed markedly different expression patterns of cytokines when compared to monoculture. Still, no difference in sensitivity to gemcitabine, paclitaxel and oxaliplatin was found, indicative of the limitations of cell line-derived cultures as translational models [39]. These limitations may include the absence of a “true” desmoplastic response, resulting from the production of collagen by activated PSCs, which previously has been demonstrated in coculture of PSCs and pancreatic cancer murine organoids by Öhlund et al. [40].

### Immune cell-, vascular- and fibroblast cocultures

A tumor property not yet implemented in pancreatic PDO models is vascularization. Vascular endothelial networks in organoid tissue models have been reported, as well as immune cells and vasculature cocultures in OrganoPlates. It is anticipated that these solutions can be applied to patient-derived biopsies [41, 42]. Another vascular model is the IMPACT platform developed by Curiochips, which has been used to investigate the effect of human umbilical endothelial cells on pancreatic cell lines [43].

The emerging coculture models may face challenges regarding clinical efficacy. For Tsai and colleagues, it took 3 weeks on average to establish CAFs, while the organoid cell culture time ranged from 3 days to 2 weeks [44]. Clinical use of these coculture models would require synchronization and reduction in establishment time of CAFs, PDOs and vasculature. An interesting finding is that the proliferation of some of the Wnt-dependent subclones from organoids cultured by Seino et al. required signaling from CAFs [31]. These findings underscore the importance of tailored culture media and stromal factors in the models, which not only mediate drug responses, but also the progression, outgrowth and composition of the organoids itself [45].

## Drug screening

### Reagent‐based assays

Efficient viability assays are crucial for the drug screening process. CellTiterGlo® 3D is the most frequently used kit for quality control and proliferation analysis of PDOs in multi-well plates. This viability assay quantifies ATP to generate a luminescent signal that is detected by a plate-reading luminometer. Driehuis and colleagues added 15 µl reagent to 384-well plates after 72 hours of drug incubation and used MG-132 and Staurosporin (pro-apoptotic agents) as positive controls [27].

Resazurin is a water-soluble dye developed to be a stable, less toxic reagent. PrestoBlue™ is a fast resazurin-based assay, where the reagent color changes upon reaction with mitochondrial enzymes along with a quantifiable shift in fluorescence. It has enough sensitivity for 384-well plates and can be used with HTS readers. With this viability assay, Bian et al. studied the effects of Bromodomain and Extra-Terminal (BET) protein inhibitors on primary organoids from 24 pancreatic cancer patients and showed that c-MYC-expression profiles could predict organoid sensitivity [46].

High-content analysis (HCA) is an automated microscopy-based screening process combining multiparametric analysis with multiple images. Jabs et al. developed an HCA workflow for organoid screening called DeathPro [47]. In this workflow, cells are stained with Hoechst (for living cells) and counterstained with propidium iodide (dead cells), and image analysis of the area of the Hoechst/propidium iodide-channels is used to calculate the drug response [47].

### Optical metabolic imaging

Walsh et al. (Skala group) reported optical metabolic imaging (OMI) as a single-cell based viability assay in pancreatic PDOs [26]. A multiphoton microscope was used to detect the fluorescence intensity of NADH over FAD to obtain the redox ratio, from which the authors calculated an OMI-index to measure metabolic activity and viability in pancreatic PDOs with high sensitivity [26]. Label-free Selective Plane Illumination Microscopy (SPIM) is another method published by the same group [48]. Here, only one plane of the sample is illuminated by a thin sheet of excitation light, which enables more rapid imaging of volumes than a multiphoton microscope. In contrast to standard viability assays, here viability can be measured after only 24 hours, and OMI has the potential to also map intra-tumoral heterogeneity, hence providing a more nuanced picture of response prediction [49]. The rationale here is that metabolic heterogeneity may be an indicator of decreased sensitivity among organoid subpopulations, which in turn worsens the outcome [49].

### Mass spectrometry

Other methods for rapid imaging include mass spectrometry. Matrigel is a protein-rich substance with a high small-molecule background signal, which poses a hurdle to mass spectrometry applications. Johnson et al. presented a microarray workflow for the application of matrix-assisted laser desorption/ionization (MALDI) mass spectrometry imaging (MSI) to PDOs [50]. Pancreatic cancer organoids were grown in Matrigel and treated with gemcitabine plus 5-fluorouracil. After drug incubation, the organoids were centrifuged at low temperature to dissociate the Matrigel. The cell pellet was transferred to a gelatin microarray, centrifuged again, and then flash-frozen for sectioning. Sections were put on standard glass slides. Intensity peaks measured with MALDI-MSI identified a significantly altered presence of ADP and AMP when comparing controls and treated organoids, but no quantifiable measure of viability could be derived from the observed drug metabolites and ADP/AMP-peaks [50].

### Liquid handling robotics

In addition to introducing more uniform and in vivo-like models, optimizing workflows and automated systems for PDO drug screening is essential for the integration of personalized therapy into pancreatic cancer care. 3D culture protocols fully automated by liquid handling robots have thus far only been established for kidney organoids [51]. The robotic instrumentation consisted of a BioMek el406™ plate washer and microplate stacker, a WellMate™ Dispenser and Stacker and a CyBio CyBi-Well Vario Workstation. A robotic pipeline using conventional machines requires a lesser number of cells for automation to work, introducing potential problems for PDO screening. However, Seppälä and colleagues were the first to show that reduced biomass does not have a significant impact on drug sensitivity for pancreatic cancer PDOs, demonstrating that automation can be of help to optimize and bridge steps in the workflow process [20].

## Drug response prediction

### Drug discovery and drug response modeling

Utilizing PDOs for large-scale drug screens does not only contribute to drug discovery, but can also be employed to find new companion biomarkers signaling the best individual treatment. By analysis of large amounts of bio-banked patient-derived material in a high-throughput fashion, studies have been able to correlate preclinical and clinical drug responses to genomic and transcriptomic profiles in PDOs, a process called pharmaco-typing. In high-throughput screening of 3D cultures, a microplate assay, scalable to a 1536-well format [34], is used for screening of serial compound dilutions at a 1000-fold range, while half maximal inhibitory concentration (IC_50_) values are obtained from the dose-response curves [34]. In addition, whole-genome sequencing (WGS) or SNP-based microarray analyses can be used to validate mutational profiles against the original tumor tissues [27, 52].

To date, several investigators have already employed pancreatic cancer organoids for the study of novel chemotherapeutics and relationships between the tumor microenvironment (TME) and therapeutic resistance [53]. Additionally, PDOs may represent a niche for the study of unresectable metastatic disease since they can be injected into the main pancreatic duct of immunodeficient mice. This technique could offer valuable insights into patient- and tumor type-specific progression, which in turn may benefit personalized medicine research [54].

Hou et al. (Tuveson lab and Scripps research institute) performed a toxicity screen of pancreatic tumor spheroids using a set of ~ 3300 NCI approved drugs on a 356-well platform [34]. Confocal microscopic analysis was performed on the assay plates to investigate spheroid structures, whereas drug response modeling included a cut-off to identify active compounds or “hits” among the large compound library [55, 56]. The cut-off is determined using an interval-based mathematical algorithm and validated using the Z-factor [55]. The subsequent dose-response analysis of 54 remaining compounds identified a dozen therapeutic agents with significant inhibitory effects amenable to further translational research [34].

While some drug response models can be used to tackle large compound libraries, others are suited for correlation of a wide range of data types with sensitivity to single chemotherapeutics or combinations with targeted agents. The TANDEM algorithm is a two-stage approach whereby upstream molecular data (e.g. somatic mutation, copy number alteration, methylation and cancer type) are modeled before downstream molecular (gene expression) data, avoiding dominance of the gene expression data [57]. Driehuis et al. (Clevers group) carried out HTS of 76 compounds against 24 pancreatic cancer organoids in 384-well assay plates. They performed WGS and RNA-sequencing of tumor samples from established biobanks, and modeled drug responses via the classical net elastic model [58] and the new TANDEM algorithm. Strong associations between mutation signatures and drug sensitivities in two combinations of targeted and chemotherapeutic drugs were found [27].

To stratify patients according to transcriptomic profiles, simple rank correlation can be performed [59, 60]. Tiriac et al. (Tuveson lab) studied 66 PDO cultures, in 13 of which mutational profiles were validated by WGS. Transcriptomic profiles were compared against the normalized area under the curve (AUC) for gemcitabine, nab-paclitaxel, irinotecan, 5-fluorouracil and oxaliplatin, and rank correlation was used to determine dependence between AUC and gene expression from the RNA-sequencing analyses. This approach generated clusters of sensitivity profiles to gemcitabine. A significantly better progression-free survival (772 vs. 373 days) was seen in patients with an “enriched” gemcitabine sensitivity profile, again indicating the utility of transcriptomic characterization for guiding personalized therapy [60], a concept that has been termed functional precision medicine [61]. The drug response models presented here are crucial for the future establishment of platforms to match genomic and transcriptomic alterations with evidence-based treatment, platforms which are already in place for a number of cancer types [62]. A hurdle that these studies face is a lack of matched clinical data. PDOs have shown a high concordance with available retrospective clinical data [27, 60], whereas prospective studies of clinical correlations in pancreatic cancer PDOs are still underway [20].

Pharmaco-typing could and has been optimized even further. Ooft and colleagues set out to develop a GR-based classifier tool to identify non-responders among colorectal cancer patients, meaning that only one compound concentration with the highest variability was required in the drug screening process [52]. GR metrics, as opposed to IC metrics, calculates drug potency on a per division basis, eliminating cell division speed as a confounder [63]. Seppälä et al. carried out a multi-institutional study with 76 patient-derived tumor tissues. The organoids retained similar drug sensitivities when the biomass was reduced to only 25 cells per well. In addition, they found that the number of passages did not alter drug sensitivity. The mean time from tissue extraction to pharmaco-typing was 49 days, compared to a mean time of 62 days between surgery and initiation of chemotherapy in their database [20]. Together, these studies hint at a potential for high-throughput automated drug screening assays of established organoids for therapeutic decision-making using only one drug concentration [52, 64].

## Personalized therapy workflow

Useful companion biomarkers, shortened assay times, increased biomimicry, and optimized viability assays and protocols are all important aspects of the pancreatic PDO model in need of further optimization. Still, knowledge of the interplay between the TME, pancreatic cancer subtypes and drug response is limited [52], which means that clinical implementation needs to be validated on a per-drug basis. Each compound will affect and be affected by the organoid model through several distinct and unelucidated mechanisms. However, with the technical solutions presented in this review as a basis, a cautious description of a future optimized clinical workflow for pancreatic cancer organoids could be as follows: (1) A less invasive novel biopsy technique is used to harvest tumor tissue with good cellularity. Transcriptomic characterization of the tumor tissue reveals a subtype requiring a specific documented culture medium composition. Establishment time is less than one week, and no fibroblast overgrowth or wild-type contamination is observed. (2) Cells are dissociated and resuspended in a bioprinter-specific hydrogel formulation and seeded with precision using laser-assisted bioprinting. The seeding is carried out on a microfluidic chip in coculture with fibroblasts and vascular endothelial cells. (3) Optical metabolic imaging is employed to investigate intra-tumoral heterogeneity prior to the introduction of chemotherapeutic compounds to each chip platform. The viability assay time is less than 24 hours. With the help of a classifier tool, only one concentration per chemotherapeutic compound is required. (4) The classifier tool is based on the observed intra-tumoral variability, the viability assay and companion biomarkers specifically developed for pancreatic cancer organoids, creating a robust response prediction. A chemotherapeutic regimen is introduced or adjusted accordingly.

## Conclusions and future directions

The use of PDOs may alleviate the struggles of drug development by expanding the in vitro testing capabilities. Future promising therapies, like photodynamic therapy and chemotherapeutics combined with CDK 4/6-inhibitors [65, 66] could benefit from organoid models. Studies of clinical correlation are ongoing, and a phase II trial of pancreatic PDOs is currently being designed [67]. Because of the limited therapeutic advances in pancreatic cancer that have so far been made, hope is being placed on translational models such as PDOs to facilitate the transition of research data to clinical practice. Here, we have presented an overview of PDO models, focusing on their application and optimization, with the overarching goal to facilitate personalized pancreatic cancer treatment.

## Data Availability

NA.

## Abbreviations

ALI: Air-liquid interface
AUC: Area under the curve
CAF: Cancer-associated fibroblast
HTS: High-throughput screening
IC_50_: Half maximal inhibitory concentration
LAB: Laser-assisted bioprinting
MALDI-MSI: Matrix-assisted laser desorption/ionization mass spectrometry imaging
OMI: Optical metabolic imaging
PDO: Patient-derived organoid
PSC: Pancreatic stellate cell
SPIM: Selective plane illumination microscopy
TME: Tumor microenvironment
WGS: Whole-genome sequencing
